# Micro-Droplet Detection Method for Measuring the Concentration of Alkaline Phosphatase-Labeled Nanoparticles in Fluorescence Microscopy

**DOI:** 10.3390/s17112685

**Published:** 2017-11-21

**Authors:** Rufeng Li, Yibei Wang, Hong Xu, Baowei Fei, Binjie Qin

**Affiliations:** 1 School of Biomedical Engineering, Shanghai Jiao Tong University, Shanghai 200240, China; brave_lee@sjtu.edu.cn (R.L.); wangyibei@sjtu.edu.cn (Y.W.); xuhong@sjtu.edu.cn (H.X.); 2 Emory University School of Medicine, Georgia Institute of Technology, Atlanta, GA 30329 USA; bfei@emory.edu

**Keywords:** fluorescence microscopy, micro-droplet, spot detection, alkaline phosphatase (AP), nanoparticles

## Abstract

This paper developed and evaluated a quantitative image analysis method to measure the concentration of the nanoparticles on which alkaline phosphatase (AP) was immobilized. These AP-labeled nanoparticles are widely used as signal markers for tagging biomolecules at nanometer and sub-nanometer scales. The AP-labeled nanoparticle concentration measurement can then be directly used to quantitatively analyze the biomolecular concentration. Micro-droplets are mono-dispersed micro-reactors that can be used to encapsulate and detect AP-labeled nanoparticles. Micro-droplets include both empty micro-droplets and fluorescent micro-droplets, while fluorescent micro-droplets are generated from the fluorescence reaction between the APs adhering to a single nanoparticle and corresponding fluorogenic substrates within droplets. By detecting micro-droplets and calculating the proportion of fluorescent micro-droplets to the overall micro-droplets, we can calculate the AP-labeled nanoparticle concentration. The proposed micro-droplet detection method includes the following steps: (1) Gaussian filtering to remove the noise of overall fluorescent targets, (2) a contrast-limited, adaptive histogram equalization processing to enhance the contrast of weakly luminescent micro-droplets, (3) an red maximizing inter-class variance thresholding method (OTSU) to segment the enhanced image for getting the binary map of the overall micro-droplets, (4) a circular Hough transform (CHT) method to detect overall micro-droplets and (5) an intensity-mean-based thresholding segmentation method to extract the fluorescent micro-droplets. The experimental results of fluorescent micro-droplet images show that the average accuracy of our micro-droplet detection method is 0.9586; the average true positive rate is 0.9502; and the average false positive rate is 0.0073. The detection method can be successfully applied to measure AP-labeled nanoparticle concentration in fluorescence microscopy.

## 1. Introduction

Advances in microscopy and fluorescence tools have pushed the quantitative biological research for biomolecules at nanometer and sub-nanometer scales [1,2,3]. Among these fluorescence tools, nanoparticles on which alkaline phosphatase (AP) was immobilized (AP-labeled nanoparticles for short) [4] are widely used as signal markers for tagging biomolecules of interest due to their stabilization and convenience for operation. Covered with a specific antibody, the AP-labeled nanoparticle can label one target biomolecule and emit a fluorescent signal by catalyzing the corresponding substrates. Therefore, the biomolecular concentration can be directly obtained by measuring the AP-labeled nanoparticle concentration. Traditional methods for AP-labeled nanoparticle concentration measurement are to divide the amount of total fluorescent signals from the AP-labeled nanoparticles by the volume of solution in the fluorescence microscopy image. However, it is difficult to count AP-labeled nanoparticles directly from fluorescent images since AP-labeled nanoparticles are too small to detect and are closely clustered. To solve this problem, a widely-used technology called the droplet microfluidics technique has been used to encapsulate the individual AP-labeled nanoparticle in monodispersed micro-droplets [5]. Micro-droplets with a similar size are water-in-oil droplets, which can be used as micro-reactors to encapsulate and detect AP-labeled nanoparticles [6]. All the micro-droplets encapsulate fluorogenic substrates, but only a small portion of micro-droplets would carry AP-labeled nanoparticles. Only the micro-droplets encapsulating AP-labeled nanoparticles will emit remarkable fluorescent signals via the enzymatic reaction between the APs and the corresponding fluorogenic substrates within droplets. We call these micro-droplets fluorescent micro-droplets and the others empty micro-droplets. However, empty micro-droplets may emit weak fluorescent signals that result from a few APs scattered within the micro-droplet in practice. Since the process of encapsulating AP-labeled nanoparticles in micro-droplets follows a random Poisson distribution [6,7], the probability of occurrence of the micro-droplets encapsulating AP-labeled nanoparticles can be obtained via the percentage of the fluorescent micro-droplets. Therefore, we can detect the proportion of fluorescent micro-droplets to the overall micro-droplets to measure the AP-labeled nanoparticle concentration. To achieve this purpose, micro-droplet detection is necessary to analyze the AP-labeled nanoparticle concentration.

The micro-droplet detection usually consists of two steps: detection of the overall micro-droplets and detection of fluorescent micro-droplets. There are certain problems involved in the detection of the overall micro-droplets. The empty micro-droplets with weak luminance are hard to detect due to the weak difference between empty micro-droplets and their surroundings. The complex noise environment in the fluorescence images may also increase the difficulties of micro-droplet detection. There are two important types of noises: the intrinsic photon noises resulting from the random nature of photon emission and the background noises caused by the detector’s electronics [8]. Moreover, the additional noises like small bright speckles and vesicles could also impede subsequent droplet detection. Furthermore, there is still a tough issue that most micro-droplets are closely connected in the fluorescence images.

Traditional fluorescent target detection methods have been reported in the literature [9,10,11]. In [12], the authors provide a thorough comparative evaluation of the most frequently-used spot detection methods. The study shows the superiority of the multiscale variance-stabilizing transform (MSVST) detector method [13] and the H-dome-based detector (HD) method [14]. The MSVST method combines the red variance stabilizing transform (VST) with the isotropic undecimated wavelet transform [13,15] and performs well in filtering mixed-Poisson-Gaussian noises and in detecting fluorescent particles. However, the bright speckles and vesicles in the image may lead to the false detection of micro-droplets. Being different from MSVST, the HD method detects spots by extracting peaks with an amplitude higher than a given height, called domes, in a Laplace-of-Gaussian (LoG) filtered or Gaussian-filtered image. Because the amplitude of the peaks in micro-droplets varies in a large range, the HD method may not work well in micro-droplet detection. To overcome this drawback, Rezatofighi et al. [16] proposed an improved method called the maximum possible height-dome method (MPHD) to adaptively extract the dome. However, it may not perform well when both the bright speckles and closely-connected micro-droplets appear in the image. To further improve the detection performance, Jaiswal et al. [17] proposed a multi-scale spot-enhancing filter method (MSSEF) to calculate the binary map, which is obtained by iteratively applying a threshold to the LoG filtered image with scale changing. This method can significantly improve the detection performance on multiple closely-connected particles. However, since the selected threshold with respect to the mean and variance of the image may be inaccurate, it may not perform well on the micro-droplet detection. Besides, Basset et al. [18,19,20] proposed methods to select the optimal LoG scale or multiple scales corresponding to the different spot sizes in the image, but test results on fluorescent micro-droplet images proved the ineffectiveness of this method for the micro-droplet detection. As explained by Smal et al. [12], most current methods follow a common detection scheme, which consists of denoising the image, enhancing the spots and, finally, extracting the target spots in a binary map to further count the micro-droplets or estimate the positions. In addition, these methods perform ineffectively for the detection of closely-connected micro-droplets by implementing a connect-component analysis method. Recently, an automatic hotspots detection framework [21] was proposed to successfully detect active areas inside cells that show changes in their calcium concentration. However, this automatic segmentation of intracellular calcium concentration in individual video frames is about 80% accurate and may not be suitable for precisely detecting a single active cell in the highly accurate concentration measurement. Therefore, there is a need to develop new approaches in order to improve the accuracy and robustness for detecting the micro-droplets.

To address these difficulties, we propose an overall micro-droplet detection method for fluorescent micro-droplet images (FMIs).

## 2. Methods

The proposed method includes the following steps: (1) The Gaussian filter first removes the noise in the red fluorescent micro-droplet image. (2) The contrast-limited adaptive histogram equalization (CLAHE) [22] method divides the whole filtered image into different blocks and adaptively adjusts the local histogram of each block to enhance the contrasts of the weak luminance regions of overall micro-droplets. (3) The red maximizing inter-class variance thresholding [23,24,25] method (OTSU) segments the enhanced image to get the binary map of the overall micro-droplets. (4) By performing on the segmented binary map, the circular Hough transform method (CHT) [26,27] perfectly detects the overall micro-droplets due to its advantage in detecting the micro-droplets that are closely connected with each other. With the combined strengths of CLAHE, OTSU thresholding and the CHT methods, our method shows significant performances on the overall micro-droplet detection. Finally, the fluorescent micro-droplet can be easily extracted via an intensity-mean-based thresholding segmentation method and be counted with the CHT method again. We have compared the performance of our method on FMIs with the performances of the state-of-the-art methods including MSVST [13], MPHD [16] and MSSEF [17]. The comparative results demonstrate that our method outperforms these state-of-the-art methods.

### 2.1. Overall Micro-Droplet Detection

**Figure 1:**
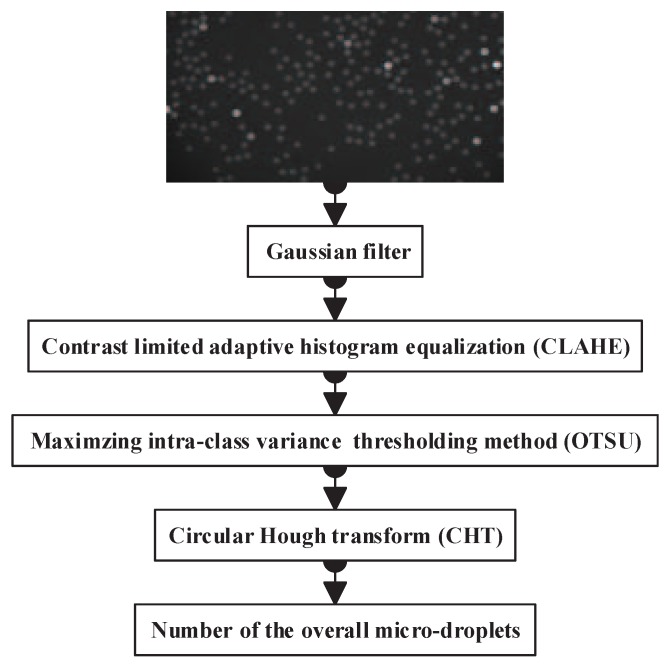
The framework of the proposed method for the overall micro-droplet detection.

Figure 1 shows the overview of the proposed method for overall micro-droplet detection. We begin by preprocessing an input image with a Gaussian filter. Then, CLAHE is performed on the local histogram of the filtered image to enhance the contrast of micro-droplets. After this image enhancement, the difference between micro-droplets and background increases, and an OTSU thresholding-based segmentation method is applied to obtain a binary map of the overall micro-droplets. Finally, the CHT method precisely detects the circular contour of the overall micro-droplets.

#### 2.1.1. Noise Reduction with the Gaussian Filter

The main noise sources in fluorescence microscopy images are the shot noise occurring in the photon counting in the imaging process and the additive Gaussian noise created by the electron characteristics of detectors [8,12]. The shot noise of the photons results from the random nature of photon emission and can be modeled as Poisson noise [8,28] when there is only a handful of photons emitted, whereas the noise can be considered as Gaussian noise when the number of photons is sufficient.

**Figure 2:**
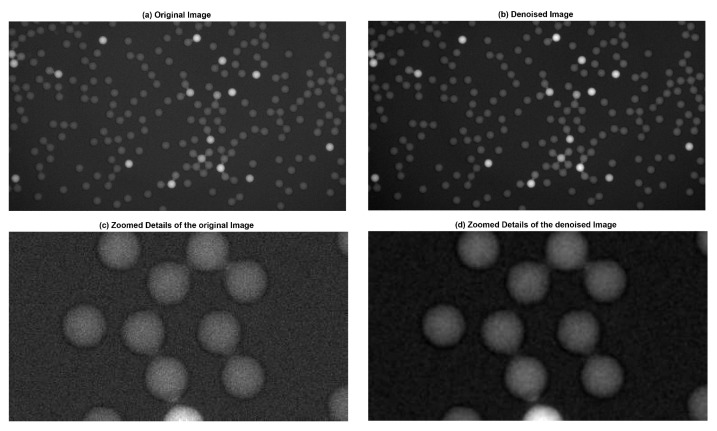
Original fluorescent image with SNR (signal-to-noise radio) of 7.1789 and the denoised image with SNR of 7.7489. (**a**) Original image. (**b**) Denoised image (*I*). (**c**) Zoomed details of the original image. (**d**) Zoomed details of the denoised image.

In most situations, the noise in fluorescent micro-droplet images can be approximately considered as Gaussian noise. Therefore, we simply use a normal Gaussian filter to remove the noise. In Figure 2, the signal-to-noise radio (SNR) of the denoised image is enhanced compared to that of the original images. We can see that the noises are eliminated effectively in the zoomed version of filtered image *I*.

#### 2.1.2. Contrast Limited Adaptive Histogram Equalization

In the filtered image, there are many micro-droplets with weak luminance. We then use the CLAHE [22] method to enhance the contrast of micro-droplets.

**Figure 3:**
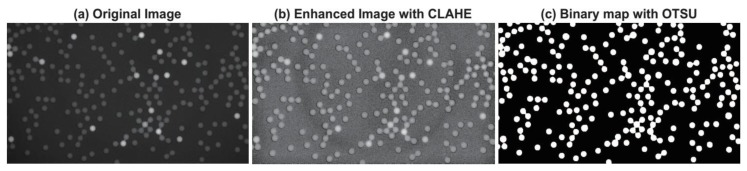
Intermediate results of contrast-limited adaptive histogram equalization (CLAHE) and OTSU on the overall micro-droplet detection: (**a**) Original image. (**b**) Enhanced image with CLAHE (*J*). (**c**) Binary map with OTSU (*K*).

Firstly, the image *I* is divided into N∗N blocks (*N* is a user-defined constant, and *N* is by default set to 8) and local histogram of every block is calculated. Since the contrast amplification in the vicinity of a given pixel value is proportional to the histogram value at that pixel value, the local histogram is clipped at a predefined value *T* to limit the over-amplification of noise. The part of the histogram exceeding *T* is redistributed among all histogram bins to keep the area of the histogram unvarying. Then, histogram equalization uses the same transformation derived from the local histogram to transform all pixels in the block and enhance local contrasts. With these operations finished, we combine all the blocks together and apply bilinear interpolation to eliminate the block effect of images. Finally, the micro-droplets at low intensities are prominently enhanced. The output of this step is an enhanced image *J*, which is shown in Figure 3.

#### 2.1.3. Maximizing inter-class Variance Thresholding Method

In Figure 3, the pixels in the enhanced image *J* can be grouped into two classes including background and micro-droplet pixels in terms of histogram distribution. Therefore, OTSU thresholding [23,24,25] is the most suited method to extract micro-droplets via histogram thresholding. The optimal threshold of this method is chosen by maximizing inter-class variance. The segmented binary map by the OTSU method effectively highlights the desired micro-droplets. However, there may be several falsely detected spots in the binary map due to the bright specks having a far smaller size than the micro-droplets in image *J*. In order to obtain accurate detection results, the morphological opening operation is used for post-processing to eliminate the influence of these abnormal spots. The output of this step is denoted as image *K*.

#### 2.1.4. Circle Detection via Circular Hough Transform

After getting the segmented binary map, we must count the number of overall micro-droplets to achieve a final detection result. The traditional fluorescent spot detection algorithms are usually based on connected component analysis (CCA). CCA-based methods perform well on detecting isolated micro-droplets, but poorly on detecting closely-connected micro-droplets. With further observation of micro-droplets, we found that all the micro-droplets appear as round spots with a similar radius. Therefore, we can employ CHT [26,27] to detect the spots with radii in a certain range. Moreover, CHT is insensitive to deformation, rotation and scaling of the circle in the image such that it can perfectly detect the incomplete round micro-droplets and closely-connected micro-droplets with lower false detection and higher accuracy. Furthermore, it has a low computational complexity, and the only parameter we need to set is the radius range of micro-droplets.

The CHT algorithm contains the following two essential steps:

- Accumulator array computation:
  The edge detection is carried out on the binary map to get an edge image (*L*). The edge pixels of *L* are designated as candidate pixels and are allowed to cast ‘votes’ in the accumulator array *A*(***a***), which represents the weight of the circle with a fixed radius and the center of the circle. Here, $a\;=\;\left\{a,b,r\right\}$. (*a, b*) represents the space location of pixels, and *r* is the radius of the expected circle. At the beginning, all the elements of *A*(***a***) are set to 0.
- Center and radius estimation:
  For every pixel ***x*** of the fluorescence image, we accumulate all the units of *A*(***a***) that satisfy the function f(x,a)=0. f(x,a) is the analytical expression of circle:
  (1) $$f\left(x,a\right)={\left(x_{1}-a\right)}^{2}+{\left(x_{2}-b\right)}^{2}-r^{2}$$
  Finally, the circular centers and radii are estimated by detecting the peaks in the accumulator array. We can get the number of micro-droplets by counting the centers of detected circles.

The overall micro-droplets can be detected with the method mentioned above. This method can accurately extract and count the overall micro-droplets on FMIs.

### 2.2. Fluorescent Micro-Droplet Detection

**Figure 4:**
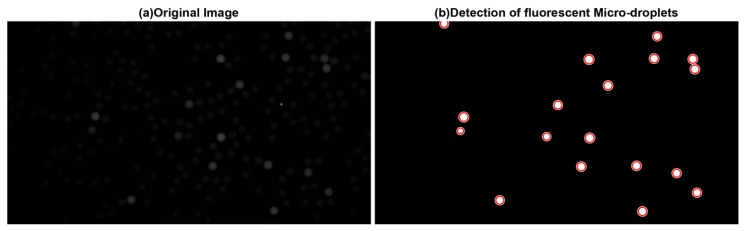
Detection of fluorescent micro-droplets. (**a**) Original image. (**b**) Detection of fluorescent micro-droplets.

The fluorescent micro-droplets can be extracted by directly thresholding segmentation due to their high intensity and round shape. However, it is difficult to choose the segmental threshold (*D*) since the fluorescent micro-droplets in different images can appear to be very different in the fluorescence intensities. We collected the manually-segmented threshold of fluorescent micro-droplets and the intensity mean of the images. By analyzing the relationship of the manually-segmented threshold and the intensity mean of the image, we found that the segmental threshold has a significant linear correlation with the intensity mean of the image. Therefore, we model the relationship mentioned above with a linear fitting method and set up a linear function corresponding to the threshold *D* of the images:

(2) D(m)=1.3717∗m+0.0126

where *m* denotes the intensity mean of the image. After the binary map is obtained, the circular Hough transform (CHT) method (see the details in Section 2.1.4) is applied to count the fluorescent micro-droplets precisely. Figure 4 shows the detected result of this method.

### 2.3. Measurement of AP-Labeled Nanoparticle Concentration

Encapsulating AP-labeled nanoparticles delivered to the droplet-generation nozzle at random is a Poisson process. The probability of encapsulating *k* AP-labeled nanoparticles in a micro-droplet is then given by equation:

(3) $$P\left(k\right)=e^{-\lambda }\frac{\lambda^{k}}{k!}$$

where λ is the average number of AP-nanoparticles per micro-droplet, *e* is the base of the natural logarithms, *k* is from natural numbers and k! is the factorial of *k*.

After detecting the numbers of fluorescent and overall micro-droplets, we can obtain the probability P(k≥1) by directly computing the proportion of the fluorescent micro-droplets to the overall micro-droplets. Then, the average number of AP-nanoparticles per micro-droplet λ can be calculated according to Equations (3) and (4):

(4) P(k=0)+P(k≥1)=1

Finally, λ is converted to the average amount of substance *n* (in moles) of a single micro-droplet; the AP-labeled nanoparticle concentration *c* is then measured by dividing the average amount of substance *n* (in moles) by the average volume *V* of a single micro-droplet. The concentration unit is given by fM, which corresponds to $10^{-15}$ mol/L.

### 2.4. Evaluation

The performance of the overall micro-droplet detection method can be evaluated in the following aspects: (1) Visual evaluations: The visual evaluations firstly give an intuitive performance comparison overview for all the detection methods. (2) TPR and FPR: The true positive rate (TPR) represents the number of true positives (TP) divided by the number of targets in ground truth data, and the false positive rate (FPR) represents the number of the false positives (FP) divided by the number of backgrounds in ground truth data. These two metrics can reflect the detection capability of an algorithm from different perspectives. (3) ROC and F-measure: For the overall evaluation of the detection method, the receiver operating characteristic (ROC curve) is used as a graph metric to uncover the detection power with different TPRs. The area under ROC (AUC) is an estimate of the area under ROC, which indicates the predictive power of the detector. Detectors with higher AUC have better detection power. Furthermore, we computed the F-measure defined by the harmonic mean of precision and recall F=2∗Prec∗Rec/(Prec+Rec). The precision metric Prec is defined as Prec=TP/(TP+FP), and Rec is the index of recall defined as Rec=TP/(TP+FN), where FN is the number of false negatives. The F-measure is a widely-used metric to measure the accuracy of the detection method. The higher F-measure score is related to the higher accuracy. (4) Overall number of micro-droplets detected: The purpose of our work is to precisely count micro-droplets. Therefore, the comparative results on the number of overall micro-droplets detected can directly reflect the superiority of our method.

The fluorescent micro-droplet detection method is evaluated by counting the number of fluorescent micro-droplets detected. We demonstrate the accuracy of this work via the relative error that is defined as the proportion of counting error to the true number counted manually. The counting error is the absolute value of the difference between the true number and the detected number. Low relative errors demonstrate high detection performances.

Finally, we calculate a test AP-labeled nanoparticle concentration with the results of micro-droplet detection and compute a reference concentration with the ground truth data. Then, we use the relative error again to compute the accuracy of our method for the AP-labeled nanoparticle concentration measurement. The comparative results of the test and the reference AP-labeled nanoparticle concentration further demonstrate the performance of our method.

### 2.5. Code

The source code for the proposed algorithm and associated MATLAB-based GUI are freely available on the author’s website, along with instructions for installation and use: http://www.escience.cn/people/bjqin/research.html.

## 3. Results

This section gives the evaluation of the proposed method on the FMIs acquired from the Nano Biomedical Research Center (NBRC) in Shanghai Jiao Tong University, China. All the FMIs are acquired using an inverted fluorescence microscope (Olympus IX73, Olympus Ltd., Tokyo, Japan) at 100-times magnification when the fluorescence is fully developed. The size of FMI in pixels is 1080×1920, and the diameter of the micro-droplets in the image is approximately 30 μm. The relative experiment details are demonstrated as below. The APs encapsulated in the micro-droplet are obtained from calf intestine. Both APs and AP-labeled nanoparticles were synthesized by NBRC. The substrate concentration employed was 5 mM, where mM represents $10^{-3}$ mol/L.

### 3.1. Overall Micro-Droplet Detection

Quantitative evaluations of our method and the state-of-the-art methods mentioned above were carried out on the FMIs. The FMI data consist of a total of fifteen test images. The ground-truth of the overall micro-droplets on the FMIs was manually segmented by two experts at NBRC.

The three methods’ parameters are set with the default parameters for achieving the best performances of these methods. As for our method, we set the stand variance σ of Gaussian filtering to one. The contrast enhancement threshold *T* is set to 0.05, and *N* is set to eight by default to make the CLAHE achieve the best performances. The search radius of circular Hough transform is set from 16 to 32. All the parameters of our method are set to make our method perform best.

#### 3.1.1. Visual Evaluation

**Figure 5:**
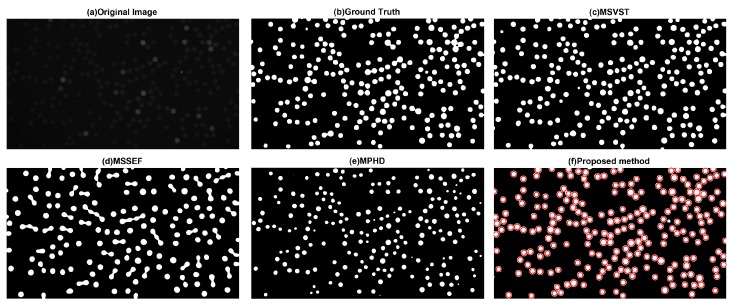
Comparative results of segmented binary maps of different methods: (**a**) Original image. (**b**) Ground truth. (**c**) Multiscale variance-stabilizing transform (MSVST). (**d**) Multiscale spot-enhancing filter method (MSSEF). (**e**) Maximum possible height-dome method (MPHD). (**f**) The proposed method.

The visual evaluation of different methods is shown in Figure 5. We can see that the proposed method (Figure 5f) perfectly detects all the micro-droplets. Figure 5c also shows that MSVST may detect false micro-droplets. Moreover, Figure 5d,e demonstrates that MSSEF and MPHD may perform poorly on the overall micro-droplet detection.

#### 3.1.2. TPR and FPR

**Figure 6:**
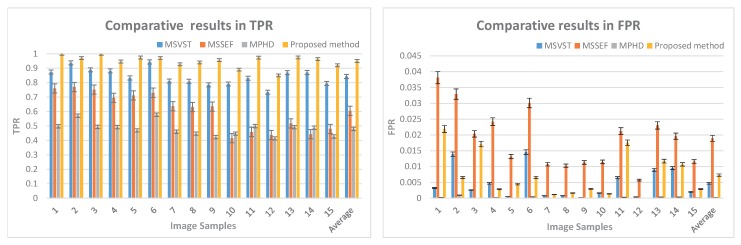
Comparison of TPR and FPR obtained with MSVST, MSSEF, MPHD and the proposed methods on fluorescent micro-droplet images (FMIs).

The comparative evaluations of TPR and FPR are displayed in Figure 6. The TPR of our method is the highest TPR for all the test images, and the highest average TPR achieved by our method is 0.9502. The average FPR of our method is 0.0073. These performance metrics prove that the proposed method has achieved a satisfying micro-droplet detection compared with other methods.

#### 3.1.3. ROC and F-Measure

**Figure 7:**
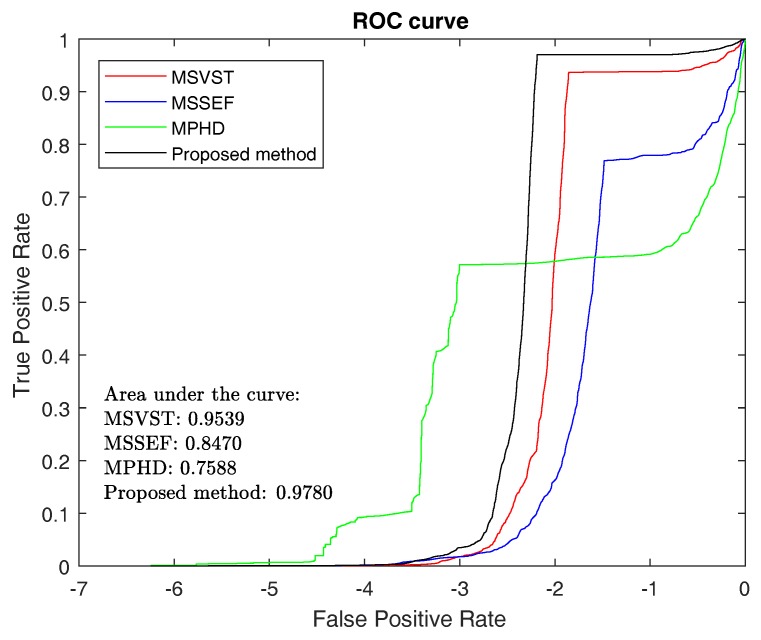
Comparison of the ROC curve obtained with MSVST, MPHD, MSSEF and the proposed methods.

The ROC curve in Figure 7 is created by plotting the TPR against the FPR at various threshold settings. Since the AUC is used to evaluate the detecting power of the method, we can use this metric to further reveal the advantage of our method. As shown in Figure 7, the AUC of the proposed method is the highest in all comparative methods. Therefore, we conclude that the proposed method has achieved the best micro-droplet detection performance.

The F-measure is usually used as a detection accuracy metric to evaluate the comprehensive performance of the detector. The higher F-measure corresponds to the better detection. The evaluation results of the F-measure are listed in Table 1. The best average detection accuracy 0.9586 is achieved by our micro-droplet detection method. This highest F-measure score verifies the superiority of the proposed method over other methods.

#### 3.1.4. Detected Number of Overall Micro-Droplets

Table 2 shows the final number of micro-droplets detected with different methods. The true number of overall micro-droplets is acquired manually by two experts. Compared with other methods, the proposed method performs stably in detecting capability of the overall micro-droplets in all 15 images, and the detected error is less than two for all the images.

### 3.2. Fluorescent Micro-Droplet Detection

The detected results of fluorescent micro-droplets are shown in Table 3. The true number of fluorescent micro-droplets is obtained manually by two experts. For the total test images, the proposed method has obtained 100 percent detection accuracy for the thirteen test images with the relative errors in detecting the remaining two images achieving 6.25% and 6.06%. These detected results demonstrate the proposed method’s capability in accurately detecting the fluorescent micro-droplets.

### 3.3. AP-Labeled Nanoparticle Concentration Measurement

Compared with the reference concentration (Table 4), the test AP-labeled nanoparticle concentration calculated with the detected results of micro-droplets has been measured with high accuracy in most samples. The low relative errors in Table 4 further demonstrate the high performance of our method in the measurement of AP-labeled nanoparticle concentration. fM in Table 4 corresponds to $10^{-15}$ mol/L.

## 4. Discussion

The comparative evaluations demonstrated in Section 3 reveal the effectiveness of the proposed method for micro-droplet detection. With the precise micro-droplet detection, the AP-labeled nanoparticle concentration for the experimental analysis can be calculated accurately. However, it should be noted that the AP-labeled nanoparticle concentration measurement is sensitive to the results of micro-droplet detection. The results in Table 2, Table 3 and Table 4 show that a very slight micro-droplet detecting error may significantly increase the AP-labeled nanoparticle concentration measurement error. Therefore, there is certainly room for further improvement of the proposed method.

## 5. Conclusions

AP-labeled nanoparticle concentration measurement is of great importance for quantitative biomolecular analysis and measurement. Because the micro-droplet can encapsulate a single AP-labeled nanoparticle and be imaged in fluorescence microscope, the AP-labeled nanoparticle concentration measurement is usually calculated by accurately counting the fluorescent micro-droplets and the overall micro-droplets. This work proposes a micro-droplet detection method for high accuracy AP-labeled nanoparticle concentration measurement by precisely and robustly detecting the weakly luminescent empty micro-droplets that are closely clustered in the complex background noises. The comparative evaluations using the state-of-the-art methods have demonstrated that the proposed method has the best accuracy for micro-droplet detection and AP-labeled nanoparticle concentration measurement.

## Figures and Tables

**Table 1:** Comparison evaluation of the F-measure obtained with MSVST, MSSEF, MPHD and the proposed methods on FMIs.

Samples	MSVST	MSSEF	MPHD	The Proposed Method
Image1	0.9204	0.7414	0.6640	**0.9231**
Image 2	0.9306	0.7889	0.7250	**0.9674**
Image 3	0.9348	0.8127	0.6610	**0.9597**
Image 4	0.9260	0.7678	0.6591	**0.9656**
Image 5	0.9075	0.8038	0.6392	**0.9770**
Image 6	0.9343	0.7737	0.7318	**0.9677**
Image 7	0.8945	0.7607	0.6311	**0.9604**
Image 8	0.8931	0.7564	0.6183	**0.9663**
Image 9	0.8792	0.7569	0.5946	**0.9721**
Image 10	0.8810	0.5775	0.6183	**0.9402**
Image 11	0.8999	0.6082	0.6653	**0.9655**
Image 12	0.8462	0.6044	0.5859	**0.9186**
Image 13	0.9177	0.6545	0.6586	**0.9707**
Image 14	0.9202	0.5954	0.6555	**0.9692**
Image 15	0.8831	0.6368	0.5987	**0.9551**
Average	0.9046	0.7093	0.6471	**0.9586**

**Table 2:** Comparison evaluation of the overall number of detected micro-droplets.

Samples	True Number	MSVST	MSSEF	MPHD	The Proposed Method
Image1	161	163	93	152	161
Image 2	222	232	142	202	222
Image 3	221	223	142	202	221
Image 4	223	227	135	198	222
Image 5	219	224	149	202	218
Image 6	229	235	152	210	229
Image 7	250	255	150	236	249
Image 8	239	245	149	224	240
Image 9	245	246	141	224	245
Image 10	381	393	155	350	381
Image 11	372	383	159	348	372
Image 12	381	386	175	345	381
Image 13	347	356	166	320	349
Image 14	414	422	175	371	412
Image 15	358	365	164	325	357

**Table 3:** Comparison evaluation of the number of detected fluorescent micro-droplets.

Samples	True Number	Detected Number of Fluorescent Micro-Droplets	Relative Error
Image1	21	21	0.00%
Image 2	18	18	0.00%
Image 3	18	18	0.00%
Image 4	16	17	6.25%
Image 5	13	13	0.00%
Image 6	24	24	0.00%
Image 7	27	27	0.00%
Image 8	26	26	0.00%
Image 9	9	9	0.00%
Image 10	36	36	0.00%
Image 11	28	28	0.00%
Image 12	30	30	0.00%
Image 13	33	35	6.06%
Image 14	32	32	0.00%
Image 15	31	31	0.00%

**Table 4:** Comparison evaluation of the alkaline phosphatase (AP)-labeled nanoparticle concentration measurement.

Samples	True AP-Labeled Nanoparticle Concentration (fM)	Test AP-Labeled Nanoparticle Concentration (fM)	Relative Error
Image1	16.4222	16.4222	0.00%
Image 2	9.9356	9.9356	0.00%
Image 3	9.9825	9.9825	0.00%
Image 4	8.7483	9.3610	7.00%
Image 5	7.1905	7.2246	0.47%
Image 6	13.0088	13.0088	0.00%
Image 7	13.4291	13.4862	0.43%
Image 8	13.5327	13.4730	0.44%
Image 9	4.3976	4.3976	0.00%
Image 10	11.6625	11.6625	0.00%
Image 11	9.1947	9.1947	0.00%
Image 12	9.6366	9.6366	0.00%
Image 13	11.7421	12.4174	5.75%
Image 14	9.4524	9.5002	0.51%
Image 15	10.6424	10.6736	0.29%
